# Storax Inhibits Caveolae-Mediated Transcytosis at Blood-Brain Barrier After Ischemic Stroke in Rats

**DOI:** 10.3389/fphar.2022.876235

**Published:** 2022-07-08

**Authors:** Min Zhou, Dongna Li, Qian Shen, Lei Gao, Pengwei Zhuang, Yanjun Zhang, Hong Guo

**Affiliations:** ^1^ Department of Traditional Chinese Medicine, Tianjin Medical University General Hospital, Tianjin, China; ^2^ Chinese Materia Medica College, Haihe Laboratory of Modern Chinese Medicine, Tianjin University of Traditional Chinese Medicine, Tianjin, China; ^3^ The Microscopy Core Facility, Westlake University, Hangzhou, China; ^4^ State Key Laboratory of Component-based Chinese Medicine, Tianjin University of Traditional Chinese Medicine, Tianjin, China

**Keywords:** ischemic stroke, blood-brain barrier, transcytosis, caveolae, storax

## Abstract

**Background and Purpose:** Blood-brain barrier (BBB) disruption following ischemic stroke (IS) contributes to hemorrhagic transformation, brain edema, increased neural dysfunction, secondary injury, and mortality. The prevailing view attributes the destruction of tight junction proteins (TJs) to the resulting BBB damage following IS. However, recent studies define a stepwise impairment of the transcellular barrier followed by the paracellular barrier which accounts for the BBB leakage in IS. The increased endothelial transcytosis that has been proven to be caveolae-mediated, preceding and independent of TJs disintegration. Emerging experimental investigations suggested Storax attenuates BBB damage after stroke. This study aimed to test our hypothesis that Storax inhibits caveolae-mediated transcytosis at BBB after ischemic stroke in rats.

**Methods:** Male Wistar rats (250–300 g) were subjected to transient middle cerebral artery occlusion (t-MCAO). Brain water content and the cerebral infarction size were assessed by brain tissue drying-wet method and 2,3,5-triphenyltetrazolium chloride (TTC) staining. BBB permeability was detected by the leakage of Evans blue and Albumin-Alexa594. The ultrastructure of BBB was examined by transmission electron microscopy (TEM). Cav-1 and Mfsd2a were quantified by western blotting and immunofluorescence staining, AQP4, PDGFR-β, ZO-1 and Occludin were quantified by western blotting.

**Results:** Storax treatment of 0.1 g/kg had no significant effects on brain lesions. Storax treatment of 0.2, 0.4, and 0.8 g/kg led to a significant decrease in infarction size, and the Storax 0.4, 0.8 g/kg groups displayed a significant reduction in brain water content. Storax treatment of 0.8 g/kg showed mild toxic reactions. Thus, 0.4 g/kg Storax was selected as the optimal dose for subsequent studies. Storax significantly inhibited the fluorescent albumin intensity in the brain parenchyma and the number of caveolae in ECs, alongside attenuating the ultrastructural disruption of BBB at 6 h after stroke. Meanwhile, Storax significantly increased the expression of Mfsd2a and PDGFR-β, and decrease the expression of Cav-1 and AQP4, corresponding to the significantly decreased Cav-1 positive cells and increased Mfsd2a positive cells. However, Storax has no significant effects on Evan blue leakage or the expression ZO-1, Occludin.

**Conclusion:** Our experimental findings demonstrate Storax treatment inhibits caveolae-mediated transcytosis at BBB in the focal stroke model of rats. We also speculate that regulation of Cav-1, Mfsd2a, AQP4, and PDGFR-β expressions might be associated with its beneficial pharmacological effect, but remain to define and elucidate in future investigation.

## Introduction

Ischemic stroke is caused by the interruption of blood supply to the brain, which leads to cerebral ischemia, anoxia, and necrosis, and it is a common disease worldwide with high mortality and disability (Powers, 2020). After ischemic stroke, cerebral ischemia and subsequent reperfusion injury result in harmful outcomes, involving oxidative stress, intracellular Ca^2+^ overload, apoptosis of nerve cells, and the breakdown of the blood-brain barrier (BBB) (Ajoolabady et al., 2021). Although several processes that cause ischemic damage to neurons inflammation, oxidative stress, and apoptosis of brain cells during ischemic stroke have been described (Jiang et al., 2021; Naseh et al., 2021), the disruption mechanisms of the BBB have not been studied as clearly as the above description at present.

BBB is a specialized structure that strictly regulates the transport of metabolites between the blood and brain substrate, and maintains the homeostatic microenvironment of the central nervous system (CNS) (Xiao et al., 2020; Zhang et al., 2020). However, the integrity and barrier function of BBB are disrupted after cerebral ischemia, leading to water and albumin through the vascular within hours (Jiang et al., 2018), and other potentially toxic and inflammatory substances in the plasma enter the brain parenchyma within several days (Yang et al., 2019). Moreover, early BBB breakdown is correlated to hemorrhagic transformation and poor clinical outcomes in cerebral ischemia (Kim et al., 2015). Traditionally, related studies have shown that two main transport pathway modes in the BBB, one is the paracellular pathway and the other is the transcellular pathway (Azarmi et al., 2020; Devraj et al., 2020). Although the effectiveness of the transcellular pathway has already exerted biological effects after ischemic stroke at 6 h, less attention has been paid to it previously (Parton, 2018). Although it has existed for 6h, there are few reports on it at present.

Caveolae is a plasma membrane invagination, which is found in mammals, and is similar to the 50–100 nm in diameter vesicles in morphology (Parton et al., 2018). Caveolins are the membrane integrin modified on the inner surface of caveolae, including Cav-1, caveolin-2 (Cav-2), and caveolin-3 (Cav-3) (Blochet et al., 2020). Recently, researchers have shown an increased interest in Cav-1 and indicated that Cav-1 is associated with signal transduction, vesicular transport, and maintenance of BBB integrity in the CNS (Gu et al., 2012; Wang and Head, 2019). In addition, Cav-1 deficiency leads to decreased AQP4 expression and impaired perivascular AQP4 coverage after ischemic stroke (Filchenko et al., 2020; Jiao-Yan et al., 2021). Although there are many reports on the outcome of Caveolae, most mechanisms have remained unclear in relation to BBB disruption. Therefore, investigating the disruption of BBB, which is induced by caveolae, and attending to the caveolae response may also become a key target of drug intervention.

For centuries, traditional Chinese medicine has played a vital role in medical care (Liu et al., 2020; Liu and Guo, 2020; Xu et al., 2020). Storax (*Liquidambar orientalis* Mill.) is one of the traditional Chinese medicines, used as the resuscitation with aromatics drugs to treat cardiovascular and cerebrovascular disease. Studies have shown that storax has the functions of inhibiting inflammatory responses and neuronal apoptosis, as well as protecting the integrity of BBB (Guo et al., 2011; Ni et al., 2011; Wang et al., 2020). In addition, our previous study showed that storax has a protective effect on cerebral ischemia through anti-oxidative stress, anti-inflammatory effects, and improved long-term outcomes of ischemic stroke (Zhou et al., 2013; Zhang et al., 2018; Zhou et al., 2021a). However, the mechanism of storax on BBB function, especially the transcellular barrier is still unclear. In this research, we explored the effect of storax on the caveolae-mediated transcytosis of the permeability at BBB in a t-MCAO rat model to investigate the effect of storax in regulating the expression of Cav-1, and to deeply interrogate its therapeutic effect and mechanism after BBB disruption.

## Materials and Methods

### Drug Administration and Animal Groups

Storax (Latin: *storax*; Greek: στύραξ, stúrax), often commercially sold as styrax, is a natural resin isolated from the wounded bark of *Liquidambar orientalis* Mill. (Asia Minor) and *Liquidambar styraciflua* L. (Central America) (Hamamelidaceae). Storax (batch No. 120931-201003, origin Indonesia) investigated in this study was purchased by the School of Traditional Chinese Medicine, Tianjin University of Traditional Chinese Medicine from Bozhou Medicinal Materials Market (Anhui, China), and tested to meet quality control of the *Chinese Pharmacopoeia*. A voucher specimen has been deposited in the Specimen Museum of traditional Chinese Medicine, Tianjin University of Traditional Chinese Medicine (voucher No.120113180803077LY).

Animals were divided randomly into Sham group: animals received pure water only; Model group: animals received 0.5% tween-80 solution after stroke; Storax 0.1, 0.2, 0.4, 0.8 groups: animals received Storax treatment at the dose of 0.1, 0.2, 0.4, 0.8 g/kg after stroke.

### Middle Cerebral Artery Occlusion Model

Male Wistar rats or C57BL/6 J mice [SPF grade, Vital River Laboratory Animal Technology Co., Ltd., Beijing, SCXK (Beijing) 2016-0006] were housed under temperature-controlled (25°C) and 12:12 light-dark cycle conditions, with free access to food and water. Rats (250–280 g) or mice (8 weeks old, 20 ± 2 g) were initially anesthetized with 3.5% isoflurane and maintained with isoflurane (2% for rats and 1% for mice) in 5% CO_2_ and 95% O_2_ by a face mask. Rectal temperature was maintained at 37 ± 0.5°C throughout the surgical procedure using a thermostat-controlled heating pad. Briefly, the right common carotid artery (CCA), external carotid artery (ECA), and internal carotid artery (ICA) were exposed. Monofilament nylon wire (0.38 ± 0.02 mm for rats and 0.20 ± 0.01 mm for mice) was inserted from the ECA and advanced into the ICA until the tip occluded the proximal stem of the middle cerebral artery (MCA), and reperfusion was achieved by withdrawal of filament after 2 h.

### Analysis of Brain Infarction Size

The brains were harvested at 24 h after t-MCAO, and subsequently, processed into six 2-mm-thick slices. Slices were incubated in 1% 2,3,5-triphenyltetrazolium chloride (TTC, Sigma-Aldrich, United States) at 37°C for 20 min and then fixed in 4% buffered formaldehyde solution for 24 h. Slices were imaged with the infarct area analyzed by the software of ImageJ and assessed brain infraction ratio based on the formula: (contralateral area-ipsilateral non-infarct area)/contralateral area ×100%.

### Assessment of Brain Water Content

The brains were harvested at 24 h after t-MCAO, the whole brain was collected and weighed, and placed into an oven at 80°C for 48 h, followed by a measurement of dried tissue weight. We assessed the brain water content based on the formula: (wet weight-dry weight)/wet weight×100%.

### Assessment of BBB Permeability

We determined whether Storax inhibits BBB permeability via observing Evans blue (EB) and Albumin-Alexa594 leakage at 6 h after stroke. Briefly, 2% EB (4 ml/kg) was injected via the tail vein at 4 h after stroke. After circulation for 2 h, rats were perfused under anesthesia. Brain tissue was collected from the ipsilateral hemisphere and homogenized by 50% trichloracetic acid and then centrifuged. The supernatant was transferred to a tube and 300 μL of ethanol was added. After thoroughly mixed, 100 μL mixture was added to 96-well plates. The absorbance was measured at 620 nm with infinite M200PRO (TECAN, Switzerland).

C57BL/6 J mice were used to detect albumin transcytosis at BBB after stroke. Mice were injected with 1% of Albumin-Alexa594 (Life Technologies, CA) into the right jugular vein and circulation for 30 min. Afterward, the brains were harvested for staining. Tissues were fixed with 4% paraformaldehyde, sectioned (10 μm coronal slices) with a cryostat and then stained with fluorescent antibodies against Glut-1 (1:1,000, Abcam, United States) to visualize brain vasculature. Sections were imaged with a fluorescence microscope (Pannoramic MIDI, Hungary). Fluorescent albumin intensity and Glut-1 intensity in the parenchyma of the brain were calculated.

### Transmission Electron Microscopy Detection

Peri-infarction regions, including the small necrotic infarct core positioning portion, were micro-dissected under a stereomicroscope. Tissues were fixed with 2% paraformaldehyde and 2.5% glutaraldehyde for 24 h and sections were then used for TEM detection. For quantitative analysis, electron micrographs of cerebral capillaries from the Model, Control, and Storax groups were analyzed using ImageJ software. Changes in tight junctions (TJs) and basement membrane (BM) structures were observed. Pericyte coverage of endothelial cells (ECs) was calculated based on the total length of inner pericyte processes around each vessel relative to the perimeter of the endothelium. The swelling ratio of mitochondria in astrocytes was measured in straight line segments at four cardinal points in the organelle. Caveolae of the endothelium were quantified by counting the number by intercepting the same area of vascular endothelium.

### Western Blotting Analysis

The brain tissue of the ipsilateral cortex was lysed in tissue lysis buffer (Beyotime, Shanghai, China). After centrifuging for 10 min at 12,000 rpm, the supernatant was obtained and used for western blotting analysis. The protein concentration was measured using BCA assay kit (Beyotime, Shanghai, China). Subsequently, the protein was separated on 10% SDS polyacrylamide gels and transferred to polyvinylidene difluoride (PVDF) membranes (Millipore, United States). After that, the membrane was incubated with primary antibodies against caveolin-1 (1:1,000, CST, United States), Mfsd2a (1:1,000, Thermo Fisher Scientific, United States), AQP4 (1:1,000, CST, United States), PDGFR-β (1:1,000, CST, United States), ZO-1 (1:1,000, Abcam, United States), Occludin (1:1,000, Invitrogen, United States), β-actin (1:5,000, Bioworld, AP0060) and GAPDH (1:10,000, Abcam, United States) at 4°C overnight. The next day, the membranes were incubated with goat anti-mouse (1:5,000, Bioss, Beijing, China) or anti-rabbit peroxidase-conjugated secondary antibody (1:5,000, Bioss, Beijing, China) for 1 h. Then, immunoreactive proteins were visualized using a chemiluminescence reagent (ECL; Millipore, United States). The staining intensity of each band was evaluated by densitometry and quantified using ImageJ software.

### Immunofluorescence Staining

Rats were perfused with cold PBS followed by 4% paraformaldehyde fixation. Brain tissues were sectioned (10 μm coronal slices) with a cryostat and then double-label immunofluorescence stained for Mfsd2a (1:200, Thermo, United States)/PECAM-1 (1:500, Santa, United States) and caveolin-1 (1:200, CST, United States)/PECAM-1 (1:500, Santa, United States) to investigate the expression of Mfsd2a and Cav-1 in the brain ECs. Images were acquired with a microscope (Pannoramic MIDI, Hungary) and processed with ImageJ software.

### Statistical Analysis

The data were analyzed by SPSS 23.0 statistical software, which was expressed as mean ± SEM. Data were tested with the Brown–Forsythe test for homogeneity of variance and the Shapiro–Wilk test for normality. One-way analysis of variance (ANOVA) followed by Bonferroni’s multiple comparison test was applied to infarction area, brain water content, BBB leakage, immunohistochemistry, capillaries ultrastructural assessment, and WB experimental data. The differences were statistically significant at *p* < 0.05.

## Results

### Storax Decreases Infarction Size and Brain Water Content at 24 h After Stroke

**FIGURE 1:**
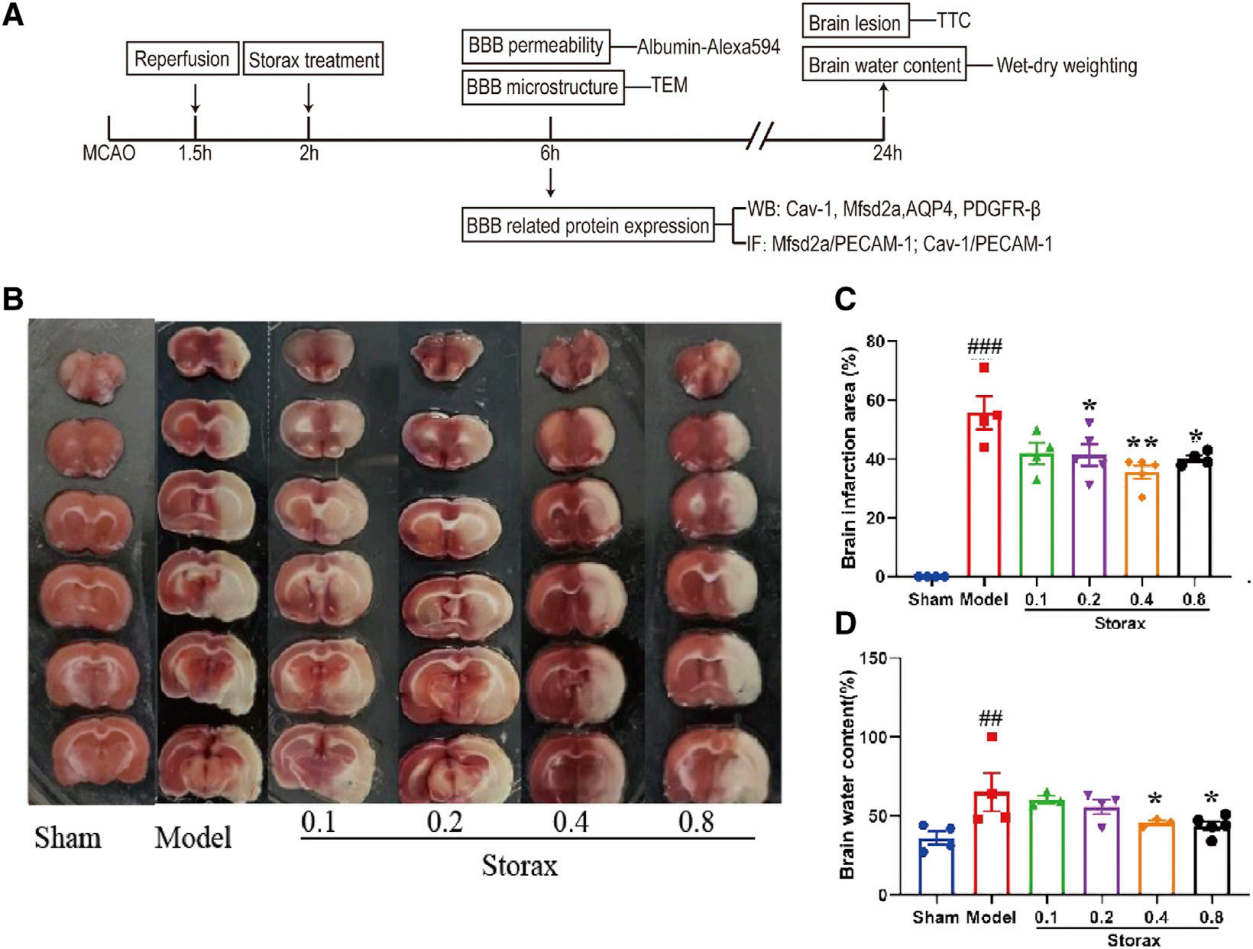
Storax decreases infarction size and brain water content at 24 h after stroke. **(A)** Schematic diagram of experimental design. MCAO: middle cerebral artery occlusion; TEM: transmission electron microscope; BBB: blood-brain barrier; IF: immunohistochemistry fluorescence; TTC: 2,3,5-triphenyltetrazolium chloride; **(B)** Representative images of TTC staining. **(C)** Quantification of the brain infarction area. **(D)** Quantification of the brain water content. Data were shown as the mean ± SEM. ^##^ *p* < 0.01, ^###^ *p* < 0.001 vs*.* Control group, *p < 0.05, **p < 0.01 vs*.* Storax group, *n* = 5.

There was no significant difference between the Model and Storax 0.1 groups. Compared to the Model, Storax 0.2, 0.4 and 0.8 g/kg treatment led to a significant decrease in infarction size [F (5, 19) = 1.489; Storax 0.2 vs. Model, *p* = 0.0046; Storax 0.4 vs. Model, *p* = 0.0040; Storax 0.8 vs. Model, *p* = 0.0454] (Figures 1A,B), and Storax 0.4, 0.8 groups displayed significant reduction [F (5, 17) = 1.553; Storax 0.4 vs. Model, *p* = 0.0474; Storax 0.8 vs. Model, *p* = 0.0163] in brain water content (Figure 1C). However, 0.8 g/kg Storax treatment showed mild toxic reactions (Supplementary Figure S1). Based on above results, 0.4 g/kg Storax was selected as the optimal dose for subsequent studies, hereinafter referred to as the Storax group.

### Storax Inhibits Albumin-Alexa594 Leakage at 6 h After Stroke

**FIGURE 2:**
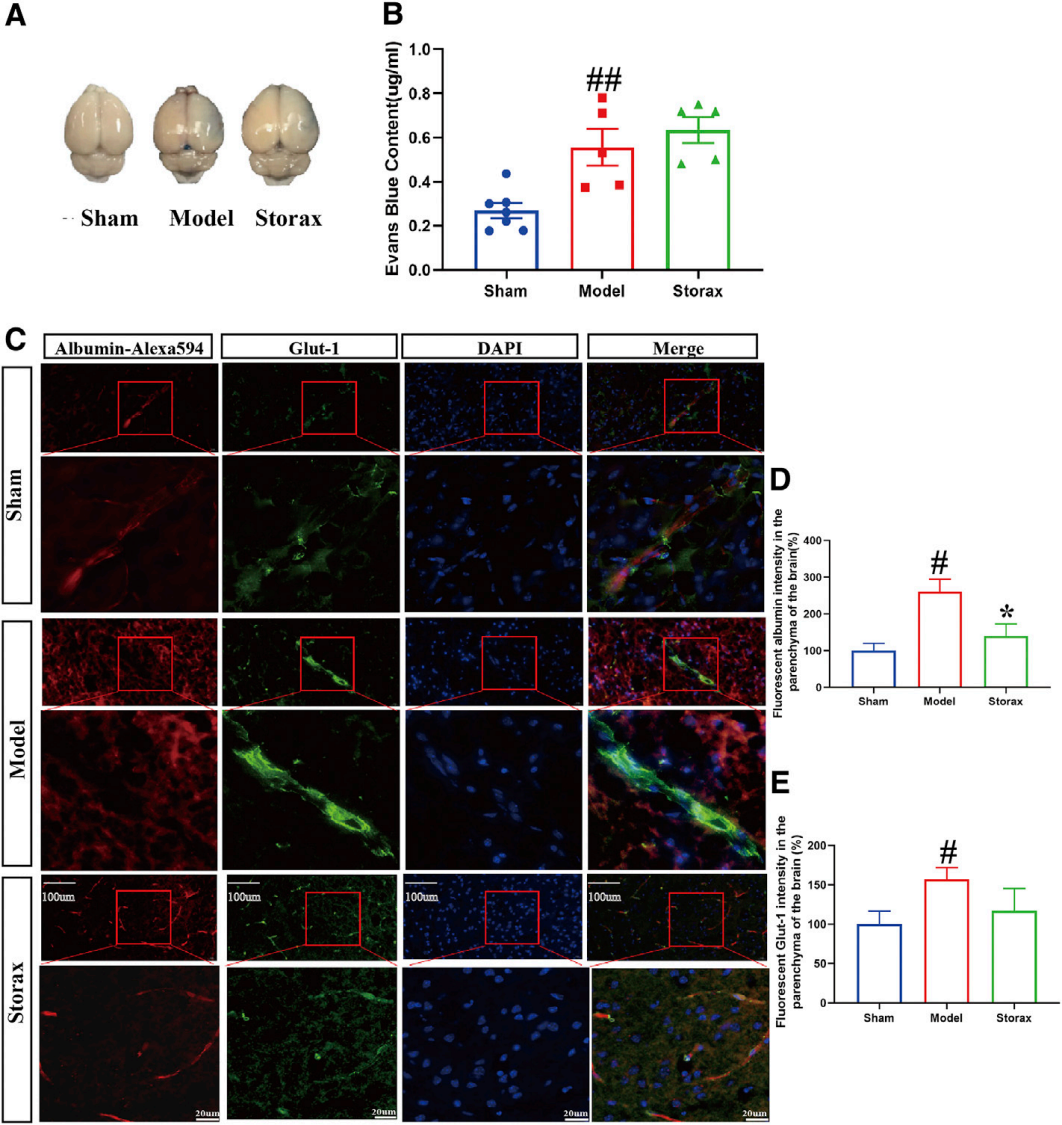
Storax inhibits Albumin-Alexa594 leakage from BBB to brain parenchyma at 6 h after stroke. **(A)** Representative images of EB staining. **(B)** Quantification of Evans blue (EB) leakage. **(C)** Representative images of Albumin-Alexa594 and Glut-1staining. **(D,E)** Quantification of fluorescent Albumin-Alexa594 and Glut-1 intensity in the parenchyma of the brain. Data were shown as the mean ± SEM. ^#^ *p* < 0.05 vs*.* Control group, *p < 0.05 vs*.* Storax group, bar = 20 μm and bar = 100 μm, *n* = 7 for EB leakage assay and *n* = 3 for Albumin-Alexa594 leakage assay.

There was no significant difference in EB leakage in the ipsilateral hemisphere between the Model and Storax 0.4 groups [F (2, 14) = 1.189; Storax vs. Model, *p =* 0.7311] (Figures 2A,B). Compared to the Control group, the fluorescent albumin [F (2, 12) = 1.546; Model vs. Control, *p* = 0.0276] and Glut-1 [F (2, 21) = 0.8236; Model vs. Control, *p* = 0.0488] intensity in the brain parenchyma of the Model group were significantly increased. Compared with the Control group, the fluorescent albumin intensity in the brain parenchyma of the ischemic boundary zone was significantly decreased [F (2, 12) = 1.546; Storax vs. Model, *p* = 0.0419] (Figures 2C–E), suggesting Storax could inhibit Albumin-Alexa594 leakage.

### Storax Inhibits Caveolae-Mediated Transcytosis and Attenuates the Ultrastructural Disruption of BBB at 6 h After Stroke

**FIGURE 3:**
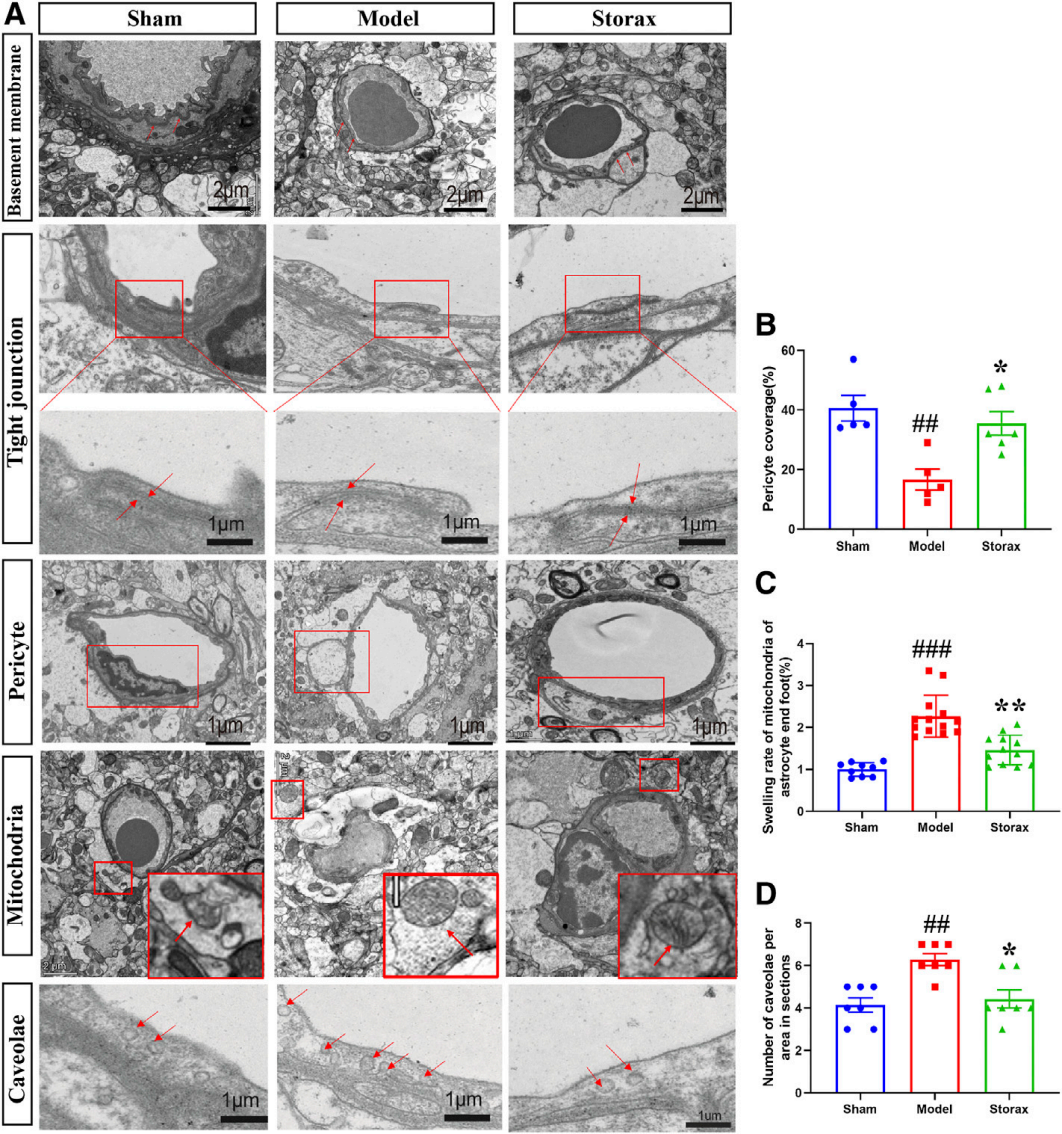
Storax inhibits caveolae-mediated transcytosis and attenuates the ultrastructural disruption of BBB at 6 h after stroke. **(A)** Representative images of the ultrastructure of basement membrane (BM), tight junctions (TJs), pericyte, astrocytic mitochondrial and caveolae in endothelial cells (ECs) under TEM observation at 6 h after stroke. **(B–D)** Quantification of the pericyte coverage, swelling rate of the astrocytic mitochondrial and the number of caveolae. Data were shown as the mean ± SEM. ^##^ *p* < 0.01, ^###^ *p* < 0.001 vs*.* Control group, *p < 0.05 vs*.* Storax group, *n* = 3.

There is no significant change between the Control and Model groups in the BM and TJs structure (Figure 3A). Compared to the Model, the average number of endothelial caveolae was significantly decreased in the Storax group [F (2, 15) = 0.3488; Storax vs. Model, *p* = 0.0046] (Figure 3D). Meanwhile, the pericyte coverage [F (2, 13) = 0.9516; Storax vs. Model, *p* = 0.0146] was significantly increased (Figure 3B) and the swelling ratio of mitochondrial in astrocytes was significantly decreased [F (2, 29) = 1.530; Storax vs. Model, *p* = 0.0004] in the Storax group. All these results suggest that Storax could inhibit caveolae-mediated transcytosis and attenuate the ultrastructural disruption of BBB.

### Effects of Storax on the Expression of BBB-Related Proteins at 6 h After Stroke

**FIGURE 4:**
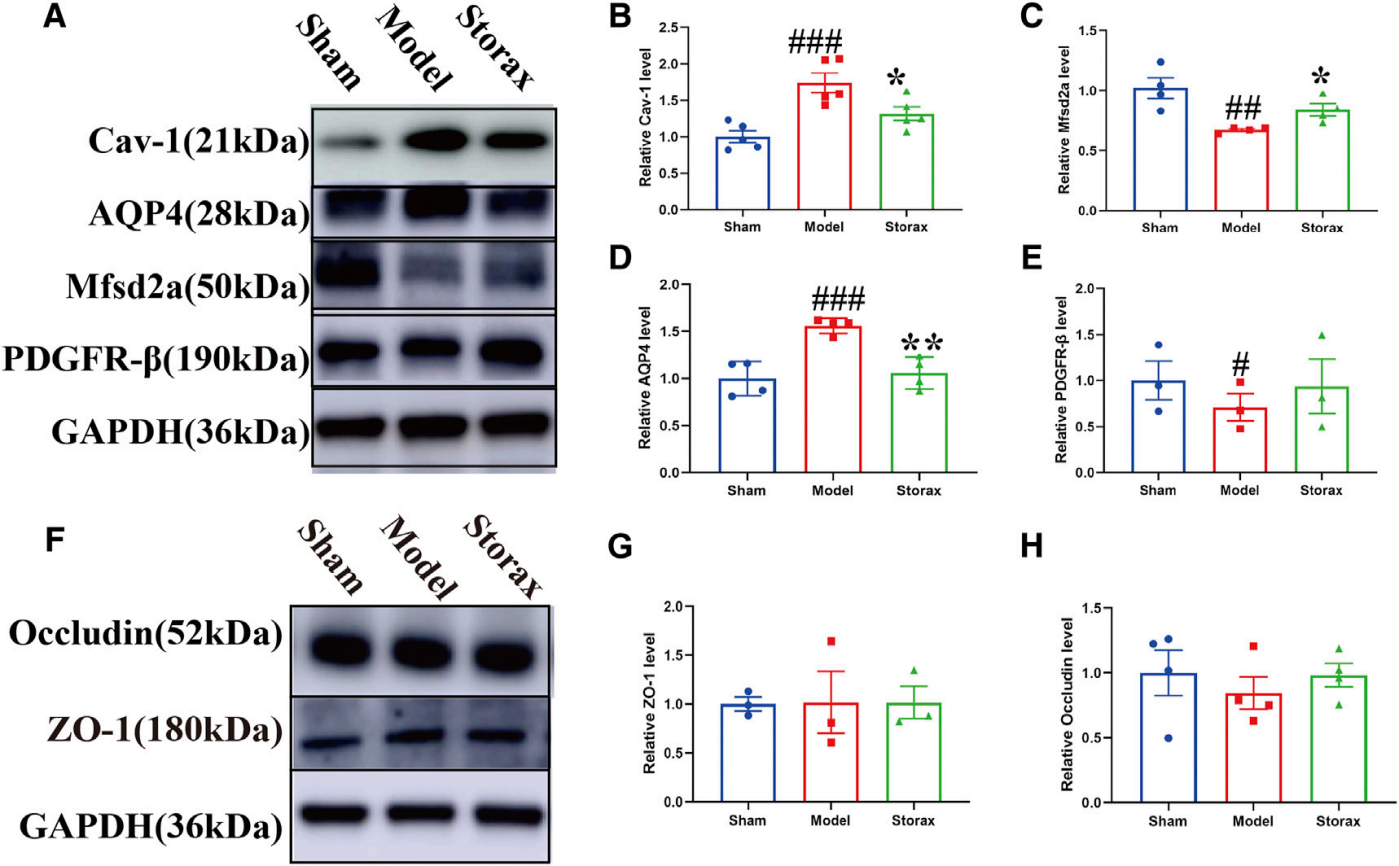
Effects of Storax on the expression of BBB-related proteins at 6 h after stroke. **(A)** Representative images of Western blotting analysis of Cav-1, AQP4, Mfsd2a and PDGF-β. **(B–E)** Quantitative data of Cav-1, AQP4, Mfsd2a and PDGFR-β expression, normalized to that of the Control group. **(F)** Representative images of Western blotting analysis of ZO-1 and Occludin. **(G,H)** Quantitative data of ZO-1 and Occludin expression, normalized to that of the Control group. Data were shown as the mean ± SEM. ^##^ *p* < 0.01, ^###^ *p* < 0.001 vs*.* Control group, *p < 0.05, **p < 0.01 vs*.* Storax group, *n* = 3–5.

To further understand the molecular mechanisms of Storax regulating caveolae-mediated transcytosis after stroke, we examined Cav-1, Mfsd2a, AQP4, and PDGFR-β expressions by Western blotting (Figure 4A). Compared with the Control group, the expression of Cav-1 [F (2, 12) = 0.3902; Model vs. Control, *p* = 0.0009] (Figure 4B), AQP-4 was significantly increased in the Model group, and the expression of Mfsd2a, PDGFR-β was significantly decreased. Compared with the Model, Storax treatment significantly downregulated the expression of Cav-1 [F (2, 12) = 0.3902; Storax vs. Model; *p* = 0.0375] and AQP-4 [F (2, 9) = 4.581; Storax vs. Model, *p* = 0.0030], and significantly upregulated the expression of Mfsd2a [F (2, 9) = 2.435; Storax vs. Model, *p* = 0.0444] and PDGFR-β [F (2, 6) = 0.3201; Storax vs. Model, *p* = 0.7670] (Figures 4B–E). Meanwhile, we also detected the expression of ZO-1 and Occludin, which are related to the paracellular pathway (Figure F). Results showed there is no significant difference in the expression of ZO-1 [F (2, 6) = 0.002368; Storax vs. Model, *p* = 0.9944] or Occludin [F (2, 9) = 0.4009; Storax vs. Model, *p* = 0.4865] among the Control, Model and Storax groups at 6 h after stroke (Figures 4G,H).

### Effects of Storax on Mfsd2a and Cav-1 in ECs at 6 h After Stroke

**FIGURE 5:**
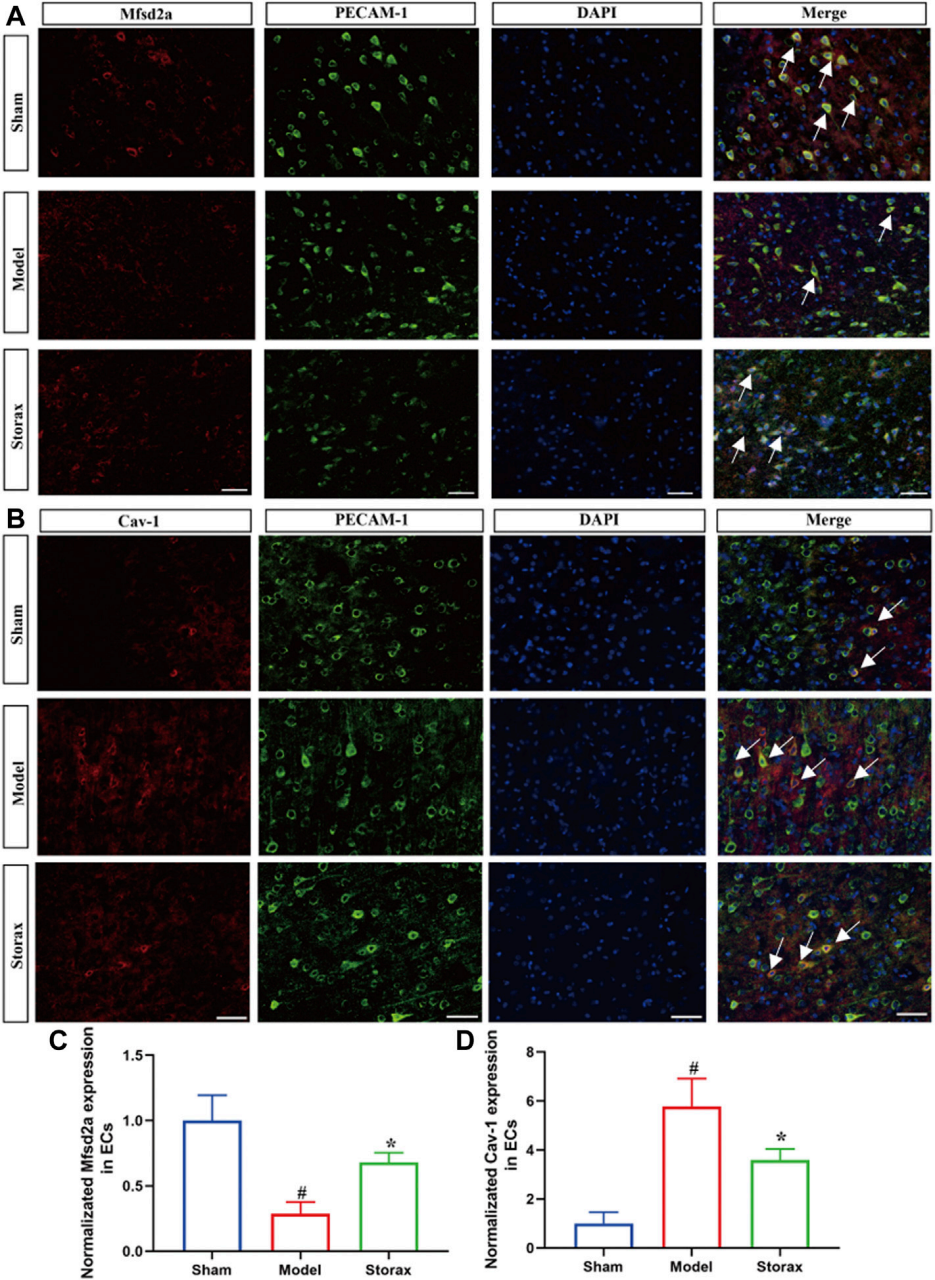
Effects of Storax on Mfsd2a and Cav-1 in ECs at 6 h after stroke. **(A)** Representative immunofluorescence images of Mfsd2a and PECAM-1. **(B)** Representative immunofluorescence images of Cav-1 and PECAM-1. **(C,D)** Quantitative data of the fluorescence intensity of Mfsd2a and Cav-1. Data were shown as the mean ± SEM. ^##^ *p* < 0.01, ^###^ *p* < 0.001 vs*.* Control group, *p < 0.05, **p < 0.01 vs*.* Storax group, bar = 50 μm, *n* = 3.

Based on the results of western blotting analysis, we further observed the effects of Storax on the expression of Mfsd2a and Cav-1 in ECs via immunofluorescence staining (Figure 5). Compared with the Control group, Mfsd2a positive cells were significantly decreased [F (2, 6) = 0.3209; Model vs. Control, *p* = 0.0198], and Cav-1 positive cells were significantly increased [F (2, 5) = 0.4655; Model vs. Control, *p* = 0.0181] in the Model group. Storax treatment significantly increased the Mfsd2a positive cells [F (2, 6) = 0.3209; Storax vs. Model, *p* = 0.0288] and significantly decreased Cav-1 positive cells (Figures 5C,D).

## Discussion

Results of the present study demonstrate that Storax alleviates brain injury of cerebral ischemia rats at 24 h after stroke, inhibits Albumin-Alexa594 leakage from BBB to the brain parenchyma, and attenuates the ultrastructural disruption of BBB at 6 h after stroke. We also found that Storax has effects in decreasing the expression of Cav-1 and AQP4 and increasing the expression of Mfsd2a and PDGFR-β at 6 h after stroke. All these data suggest Storax could inhibit caveolae-mediated transcytosis at BBB after ischemic stroke, and it might be a therapeutic candidate for treating acute BBB disruption after ischemic stroke.

Caveolae-mediated transcytosis plays an important role in cerebral ischemia (Zhou et al., 2021b). Cav-1 deficiency only significantly reduced the amount of circulating albumin transported into the brain parenchyma with endosome vesicles routed, but no effects on biocytin-TMR or IgG transportation were detected (Knowland et al., 2014). In this study, the Albumin-Alexa594 assay was used to detect the BBB permeability and endocytosis. The results showed that Storax could significantly decrease the Albumin-Alexa594 in the brain parenchyma. However, compared to the Model group, there is no significant effect of Storax on EB leakage. We speculate that it may be because a portion of EB exists as a free dye which may permeate the BBB via a paracellular pathway, and EB does not specifically bind to albumin, as pointed out in the review (Saunders et al., 2015), leading to the discrepancy between the effect of Storax on Albumin-Alexa594 and EB leakage into the brain. However, our experimental results at least proved that Storax inhibits caveolae-mediated transcytosis at BBB after ischemic stroke in rats, and this interesting discrepancy deserves further study and will be part of our next project.

The destruction of the BBB after cerebral ischemia is complex. BBB disruption occurs early and continues after reperfusion of the cerebral ischemia disease (Liebner et al., 2018). Although previous studies have shown that BBB disruption after ischemic stroke is associated with induction of inflammation, and downregulation of TJs proteins (Braidy et al., 2019; Stamova et al., 2019). It has recently become clear that the caveolae-mediated transcytosis appears to play an essential role in the early stages of cerebral ischemia, and may cause aggravation of edema formation finally (Abdullahi et al., 2018; Huang et al., 2018; Lv et al., 2018). Caveolae are widely exist in mammalian eukaryotic cells, and their structure looks like the bulb-shaped invagination observed under TEM. Moreover, some researchers revealed that BBB has already disrupted at about 3 h after ischemia, which is mainly related to the caveolae protein, such as Cav-1 response rapidly (Knowland et al., 2014; Nahirney et al., 2016). Overactivation of caveolae-mediated endocytosis led to the breakdown of BBB in the aged brain of stroke patients, early BBB disruption is associated with hemorrhagic transformation and poor clinical outcomes (Hagemann et al., 2020). In our study, the TEM showed that at the stage of 6 h after cerebral ischemia, the transcellular barrier-related structure caveolae increasingly and indicated that the transcellular permeability of BBB increases. This finding is, in part, in line with previous reports (Knowland et al., 2014; Sadeghian et al., 2018).

An increasing number of studies had reported a close correlation between storax and BBB after ischemic stroke (Ni et al., 2011; Wang et al., 2020; Xie et al., 2021). However, the shared underlying molecular mechanism of storax on BBB remained unclear. Cav-1, Mfsd2a, AQP4, and PDFGR-β were responsible for the potential shared comprehensive mechanisms in BBB. More recent research showed that loss of Cav-1 results in decreased AQP4 expression and impaired perivascular AQP4 covering after cerebral ischemia associated with altered reactive astrocyte morphology and enhanced brain swelling (Filchenko et al., 2020). In addition, Cav-1 regulates the molecule of PDGFR-β, ZO-1, and Occludin to exert effect (Hammarsten et al., 2016; Atış et al., 2019). In our study, western blotting assay was used to detect the potential of molecular mechanism. The results illustrated that the major transcellular proteins, Cav-1, Mfsd2a, and AQP4, have been changed and led to increasing transcellular permeability at the early stage of ischemic stroke, while the TJ proteins are not significantly altered or dysregulated. For the first time, we found that storax can regulate transcellular barrier proteins to protect the integrity of BBB.

Because brain ECs are the most important compartment of BBB, the protein expression in ECs are key therapeutic targets of BBB. Mfsd2a is a critical transmembrane liquid transporter and mediates DHA transport in the form of a lysophospholipid across the BBB (Stamova et al., 2019; Hagemann et al., 2020). Correlational research demonstrated that the Mfsd2a is involved in the dynamic changes of brain microvascular ECs (Knowland et al., 2014; Andreone et al., 2017; Zhou et al., 2019). Mfsd2a can modulate the neurological function such as mediating neurovascular coupling, downregulation of Mfsd2a may contribute to BBB disruption and suppresses caveolae to ensure BBB integrity (Chow et al., 2020; Zhao et al., 2020). According to recent research, Mfsd2a is an upstream regulator of Cav-1 and has been identified as an important component of BBB formation and integrity (Eser Ocak et al., 2020). In our research, we confirmed that storax attenuated BBB disruption by upregulating Mfsd2a and inhibiting Cav-1 in the ECs, to arrest the progression of cerebral ischemia.

The present work has several caveats and limitations. Firstly, we examined Albumin leakage from BBB to brain parenchyma in t-MCAO model mice but not rats. We acknowledge that consistent use of rats or mice will strengthen the evidence and will improve this problem in future studies. Secondly, it seems there is a conflict only observing significant changes in albumin leakage, but no changes in EB leakage. We are aware BBB function should also be tested for different molecular weights permeability using Sodium Fluorescein (NaF) or FITC-dextran of different molecular weights. Thirdly, Storax is a natural medicine that contains many ingredients. Further experiments to determine the active ingredients will be of great significance.

**FIGURE 6:**
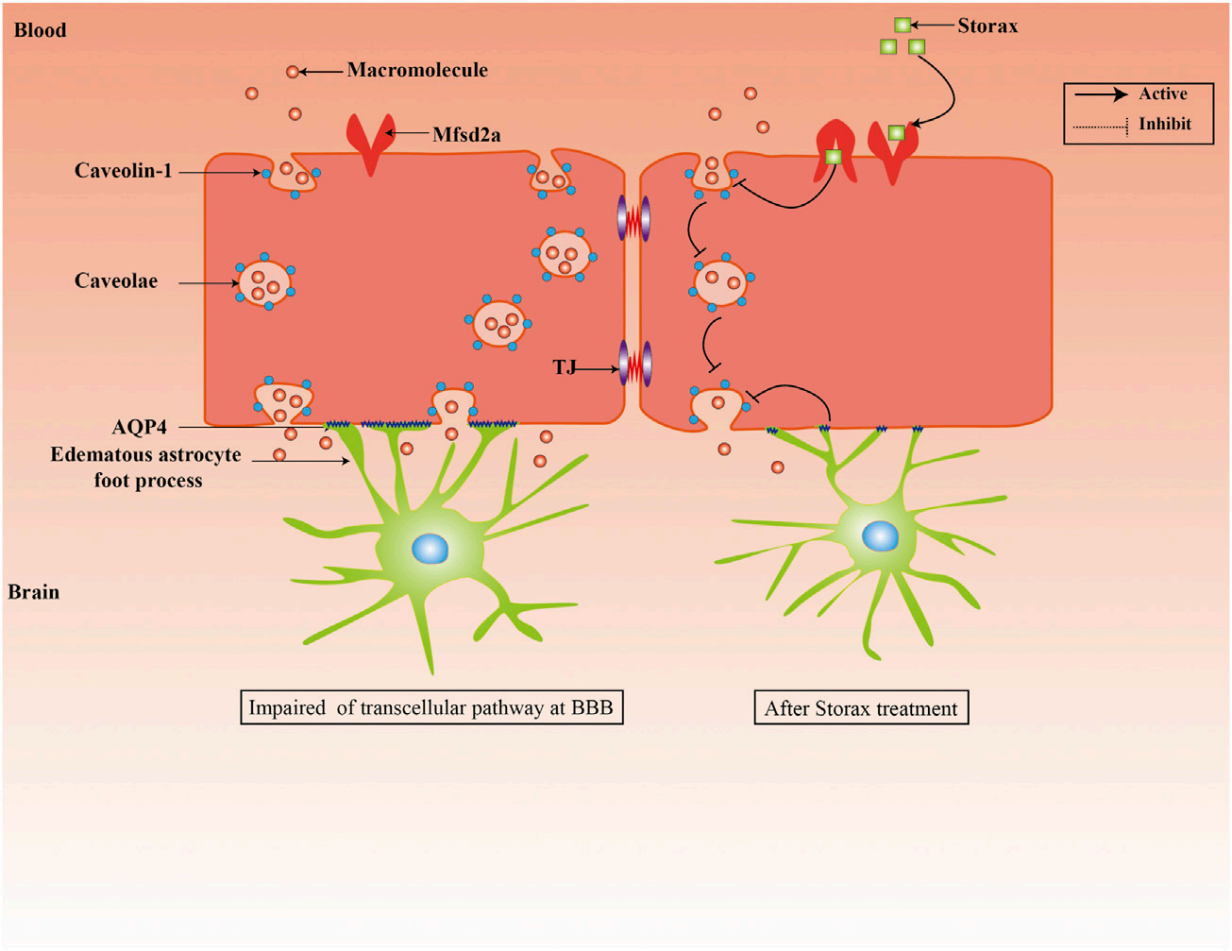
Schematic representation of effects of Storax on caveolae-mediated transcytosis at BBB and its potential molecular mechanisms.

Collectively, our study demonstrates that treatment with Storax can inhibit caveolae-mediated transcytosis at BBB after ischemic stroke in rats (Figure 6). Future investigations will demonstrate the underlying molecular mechanisms of Storax on transcytosis at BBB with space-time dynamic change, identify the key active ingredients of Storax, and evaluate the translational aspects in more rigorously designed preclinical studies.

## Data Availability

The original contributions presented in the study are included in the article/Supplementary Material, further inquiries can be directed to the corresponding authors.
