# Supplementary material for: Storax Inhibits Caveolae-Mediated Transcytosis at Blood-Brain Barrier After Ischemic Stroke in Rats
Source: Front Pharmacol. 2022 Jul 8;13:876235. doi: 10.3389/fphar.2022.876235 (PMC9304983; doi:10.3389/fphar.2022.876235)
Supplement: Supplementary file 1 [file Table1.DOCX]

**Fig. Supplement**


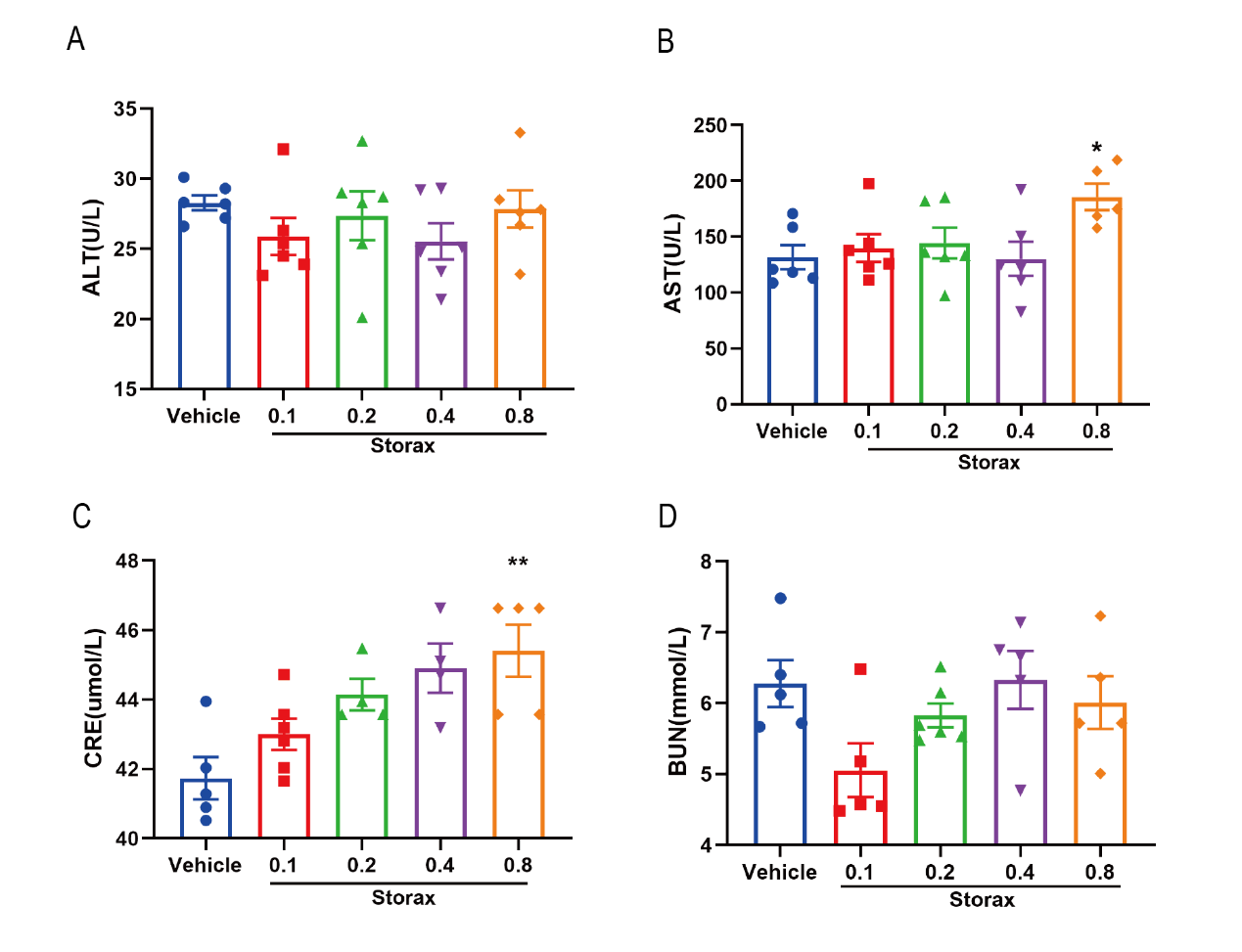


(A-D) Liver and kidney function examination. Data were shown as the mean ± SD. ^##^*P*<0.01, ^###^*P*<0.001 vs. Sham group, **P*<0.05, ***P*<0.01 vs. Storax group, n=5.
